# Direct evidence for crossover and chromatid interference in meiosis of two plant hybrids (*Lolium multiflorum×Festuca pratensis* and *Allium cepa×A. roylei*)

**DOI:** 10.1093/jxb/eraa455

**Published:** 2020-10-08

**Authors:** Marco Tulio Mendes Ferreira, Marek Glombik, Kateřina Perničková, Martin Duchoslav, Olga Scholten, Miroslava Karafiátová, Vania Helena Techio, Jaroslav Doležel, Adam J Lukaszewski, David Kopecký

**Affiliations:** 1 Institute of Experimental Botany, Czech Academy of Sciences, Centre of the Region Haná for Biotechnological and Agricultural Research, Olomouc, Czech Republic; 2 Department of Biology, Federal University of Lavras, Lavras-MG, Brazil; 3 National Centre for Biomolecular Research, Faculty of Science, Masaryk University, Kotlarska, Brno, Czech Republic; 4 Department of Botany, Faculty of Science, Palacký University, Olomouc, Czech Republic; 5 Plant Breeding, Wageningen University & Research, Wageningen, The Netherlands; 6 Department of Botany and Plant Sciences, University of California, Riverside, CA, USA; 7 University of Nottingham, UK

**Keywords:** Centromere, chromatid interference, crossover interference, homoeologous chromosome, hybrid, meiosis, recombination

## Abstract

Crossing over, in addition to its strictly genetic role, also performs a critical mechanical function, by bonding homologues in meiosis. Hence, it is responsible for an orderly reduction of the chromosome number. As such, it is strictly controlled in frequency and distribution. The well-known crossover control is positive crossover interference which reduces the probability of a crossover in the vicinity of an already formed crossover. A poorly studied aspect of the control is chromatid interference. Such analyses are possible in very few organisms as they require observation of all four products of a single meiosis. Here, we provide direct evidence of chromatid interference. Using *in situ* probing in two interspecific plant hybrids (*Lolium multiflorum×Festuca pratensis* and *Allium cepa×A. roylei*) during anaphase I, we demonstrate that the involvement of four chromatids in double crossovers is significantly more frequent than expected (64% versus 25%). We also provide a physical measure of the crossover interference distance, covering ~30–40% of the relative chromosome arm length, and show that the centromere acts as a barrier for crossover interference. The two arms of a chromosome appear to act as independent units in the process of crossing over. Chromatid interference has to be seriously addressed in genetic mapping approaches and further studies.

## Teaser

We provide direct observational evidence for chromatid interference, as well as a physical measure of the crossover interference in meiosis in plants.

## Introduction

Meiotic division is an integral part of sexual reproduction; it maintains stable chromosome numbers over generations. Chiasmata, the cytological expression of crossing over, form mechanical connections between homologous chromosomes during the first meiotic division and hence are essential elements of an orderly reduction of the chromosome number. As such, crossovers are under strict genetic control, for both their number and distribution. This control is exercised via positive crossover interference which, in essence, has a suppressive effect on the formation of another crossover in the vicinity of an already established crossover. On the other hand, a mechanism of crossover assurance guarantees that each pair of homologues gets at least one, the so-called obligatory crossover (Jones, 1984; John, 1990; Hillers, 2004; Jones and Franklin, 2006). A single crossover between two homologues ensures their normal behaviour in meiosis. The number of potential sites of crossing over, represented by double strand breaks (DSBs), is always far greater than the number of eventual crossovers, and is presumably regulated genetically and epigenetically at the whole-genome level (Wang and Copenhaver, 2018; Modliszewski *et al.*, 2018). Because of the tight control, the number of crossovers per bivalent varies between one and three for a majority of species, and only loosely correlates with chromosome length, offering more evidence for the regulation of crossover numbers (Mather, 1938; Fernandes *et al.*, 2018; Otto and Payseur, 2019).

Crossover interference was observed and described already in the very first genetic mapping experiments in *Drosophila melanogaster* (Sturtevant, 1913; Muller, 1916) and is defined as a situation where the formation of one crossover reduces the probability of another crossover in its vicinity. Its existence can be readily verified in any genetic mapping exercise because the frequency of double crossovers between any sufficiently close loci on a chromosome is always lower than the product of frequencies of independent crossovers in these segments. The frequency of double crossovers is lower the closer the monitored intervals are to each other. As a consequence, crossover events are not randomly distributed along chromosomes (Portin, 2012; Zickler and Kleckner, 2016). Generally, the extent of crossover interference decreases with distance, understood in genetic and not physical terms. In polyploids such as wheat, genetic lengths of homoeologous chromosomes (their genetic maps) are essentially the same despite large differences in their length/DNA content, demonstrating that double crossovers, when they occur, are formed much more closely physically in shorter chromosomes than in longer chromosomes. Instances of negative crossover interference have been observed frequently in lower organisms such as fungi, but at times also in certain chromosome variants in *Drosophila*, barley, and maize (Auger and Sheridan, 2001; Esch and Weber, 2002). Whether negative interference exists in higher organisms with normal chromosome structure is debatable (Säll and Bengtsson, 1989). Widespread strong negative interference was invoked in genetic mapping in tetraploid wheat (Peng *et al.*, 2000) but, with genetic maps roughly twice as long as can reasonably be expected based on chiasma frequencies and mapping in very similar populations (Lukaszewski and Curtis, 1993), marker scoring errors appear as an equally plausible explanation. Similarly, the issue of the independence of the two arms of a chromosome for crossover formation has not yet been resolved satisfactorily, and crossover interference across the centromere remains a point of dispute, with some evidence pointing in opposite directions (Laurie and Hultén, 1985; Zhao *et al.*, 1995a; Berchowitz and Copenhaver, 2010).

The mechanism of crossover interference remains unclear, but probably involves some crossover-discouraging signal or substance that spreads along the chromosome arm. The mechanical stress model of crossover interference is based on the presumption that the stress drives formation of a crossover, but in doing so the local area is then relieved and there is not enough stress to drive formation of a second crossover nearby (Börner *et al.*, 2004; Kleckner *et al.*, 2004). The polymerization model assumes that once the crossover structure is attached along the synaptonemal complex, it has the same chance per unit time to initiate a bidirectional polymerization event. The structures responsible for it are presumably the late recombination nodules which can be observed in pachytene (King and Mortimer, 1990). Crossover interference clearly involves the synaptonemal complex as it is not transmitted via synaptonemal complex discontinuities, such as synaptic partner exchange points in translocation heterozygotes, or across separated centromeres in double ditelocentric lines (reviewed in Dawe, 1998). Interestingly, there appear to be two pathways leading to crossovers, one interference sensitive and the second interference insensitive (Osman *et al.*, 2011; Mercier *et al.*, 2005). In plants, the former is more abundant and accounts for ~80–85% of total crossovers (Berchowitz *et al.*, 2007).

A bivalent in the first meiotic division is composed of two pairs of sister chromatids. Additional crossovers in a chromosome (or a chromosome arm) bring up the issue of chromatid choice for each event. It is generally accepted that for any given crossover, the chromatid choice is random, and hence a crossover involving two non-sister chromatids does not affect the choice of non-sister chromatids for another crossover in the same chromosome (Zhao *et al.*, 1995a). This is, however, an assumption with very limited experimental support, particularly in higher organisms. Direct tests of chromatid interference require analyses of all four products of any given meiosis. This condition is met only in a very few instances, all in lower organisms, such as *Aspergillus* spp. where chromatid interference does not appear to operate (Whitehouse, 1958). In higher organisms, re-analysis of experimental data from several organisms (Zhao *et al.*, 1995b) and mathematical modelling of genetic mapping data suggest that chromatid interference may exist (Teuscher *et al.*, 2000), but no direct observation has been possible thus far.

Crossover and chromatid interference can be analysed in several different ways. Generally, the former can be studied directly, and copious raw data for such analyses are generated in each genetic mapping effort, especially when mapped loci can be directly placed on the DNA sequence assemblies. Chromatid interference, on the other hand, in a great majority of organisms can be studied only indirectly, such as by mathematical modelling (Teuscher *et al.*, 2000) mentioned above. However, recent technological advances offer a chance to study both phenomena directly by cytological observation in meiosis. One such approach is to visualize sites of crossovers in meiotic cells with fluorescently labelled probes, for example for the MLH1 mismatch repair protein (Barlow and Hultén, 1998; Phillips *et al.*, 2013; Anderson, 2014). This approach, however, may lack the resolution of individual chromatids. Another way is the analysis of the anaphase I chromosomes in hybrids where the DNA sequence divergence between the parental genomes permits chromosome painting, hence direct visualization of the crossover points. Thus, it is possible not only to score the overall level and distribution of crossovers in individual chromosome arms, chromosomes, and entire genomes, but also to study their distribution among chromatids. Another advantage of this approach is that the results are not biased by potential gametophytic selection, which may distort the ratios of recombinant and parental chromosomes transmitted to the progeny. Its weakness is in the selection of parents: these need to be sufficiently different at the DNA sequence level to enable unambiguous discrimination of homologues by chromosome painting, and yet sufficiently close genetically to provide for regular chromosome pairing and disjunction. Such hybrids do exist among plant species. Here we focus on the F_1_ hybrids between representatives of *Lolium* and *Festuca*, known for a high (87–97%) metaphase I chromosome pairing and crossing over (Jauhar, 1975; Kopecký *et al.*, 2008). Scoring crossovers in anaphase I, where individual chromatids are clearly visible, offers direct evidence not only for crossover interference but also for chromatid interference and possible effects across the centromere. Cytogenetic stocks from our past experiments (Kopecky *et al.*, 2008) made it possible to analyse the physical attributes of crossing over in individual chromosomes and their arms, while high-density chromosome genetic maps (Kopecky *et al.*, 2010) made it possible to relate these observations to the DNA sequence across the entire genome. Similar observations were made on wide hybrids in the genus *Allium* (*A. cepa×A. roylei*) displaying frequent pairing and recombination between homoeologous chromosomes (Khrustaleva and Kik, 2000).

## Materials and methods

### Plant material and chromosome preparations

The frequency of possible types of crossover configurations during meiosis was evaluated in diploid F_1_ hybrids of *Festuca pratensis*×*Lolium multiflorum*. Chromosome substitution lines of *F. pratensis/L. multiflorum* hybrids were used to estimate the frequency and distribution of double crossovers for all seven *F. pratensis* chromosomes individually. The tetraploid monosomic substitution lines (2*n*=4*x*=28; 27L+1F) were developed in previous studies (Kopecký *et al.*, 2008, 2010). Plants with the same substituted *F. pratensis* chromosome were intercrossed and their progenies were germinated and individually analysed. Similarly, the F_1_ hybrids of *A. cepa×A. roylei* (Scholten *et al.*, 2016) were intercrossed and their progenies were individually screened by genomic *in situ* hybridization. Individual anthers confirmed to be in meiotic anaphase I stage and root tips were fixed in Carnoy’s solution (absolute ethanol/glacial acetic acid, 3:1 v/v) at 37 °C for 7 d and microscope preparations were made according to Masoudi-Nejad *et al.* (2002).

### Genomic *in situ* hybridization

Genomic *in situ* hybridization analyses were done on mitotic and meiotic chromosome spreads according to Kopecký *et al.* (2008). Total genomic DNA (gDNA) of *L. multiflorum* and *A. cepa* was used as blocking DNA and total gDNAs of *F. pratensis* and *A. roylei* were labelled with digoxigenin (DIG) using the DIG-Nick Translation Kit (Roche Applied Science, USA) according to the manufacturer’s instructions, and used as probes for grass and onion hybrids, respectively. The probe/blocking DNA ratio was ~1:150. Signal detection was made with anti-DIG–fluorescein isothiocyanate (FITC) conjugate (Roche Applied Science). Chromosomes were counterstained with DAPI in Vectashield (Vector Laboratories, Oberkochen, Germany). Chromosome analysis was done under an Olympus AX70 microscope equipped with epifluorescence and a SensiCam B/W camera. Images were captured with Micro Image and processed with Adobe Photoshop v.6 software.

### Scoring and statistical treatment

All statistical analyses were done in R 3.4.3 (R Core Team, 2019). For meiotic analyses of diploid F_1_ hybrids of *L. multiflorum×F. pratensis*, we evaluated exclusively the anaphase I stage. Each cell has seven pairs of chromosomes. Each arm was evaluated separately. Each pair of homologous arms displayed either no crossover or one of the possible crossover exchanges: single crossover or double crossover with two, three, or four chromatids involved. Theoretically, with no chromatid interference, the proportion of the three crossover types should be 1:2:1. We statistically treated the observed frequencies for this ratio using the multinomial test from the ‘stats’ library (R Core Team). Proportions of different numbers of crossovers on one arm between chromosomes with one and two crossovers on the other arm were compared using the two-sided Fisher’s exact test. For calculation, the function Fisher.test with *P*-values computed by Monte Carlo simulations based on 2000 replicates from the ‘stats’ library was used.

To determine the positions of the crossover events among the progenies of onion and grass hybrids, we measured the lengths of the introgressed segments and the lengths of both arms of recombined chromosomes using ScionImage software. Calculations of the distances (in megabases) between two crossovers on one arm were done based on the length of individual *F. pratensis* chromosomes (Kopecký *et al.*, 2010). The difference in distribution of different crossover types was evaluated by comparing their distributions along chromosome arms divided into 10 segments (bins) of 10% of their length. Two empirical distributions were compared using the function ks.boot in the ‘Matching’ library in R. The function uses a bootstrap version of the Kolmogorov–Smirnov test, providing accurate coverage even when the distributions being compared are not entirely continuous and ties occur in the dataset (Sekhon, 2011).

## Results

### Frequency of crossovers in anaphase I configurations in *Lolium×Festuca* hybrids

Using *in situ* probing with labelled total gDNA, we monitored crossover events involving individual chromatids in each chromosome of 96 meiocytes of *L. multiflorum*×*F. pratensis* (2*n*=2*x*=14; seven *L. multiflorum* chromosomes+seven *F. pratensis* chromosomes) F_1_ hybrids in anaphase I of meiosis. All crossovers involved chromosomes of *L. multiflorum* and *F. pratensis* and were therefore exclusively homoeologous. In total, there were 672 chromosome pairs and, subsequently, 1344 pairs of chromosome arms. Single crossovers were observed in 308 chromosome pairs and two crossovers (each on one arm) in 66 chromosomes pairs, while double crossovers (two crossovers in one arm) were observed in 133 chromosome pairs. Chromosome pairs with double crossovers in one arm and a single crossover in the other arm were observed in 26 cases. Double crossovers on both arms were observed in only six chromosome pairs. The remaining 133 pairs of chromosomes (19.8%) had no detectable crossovers.

### Direct evidence of chromatid interference

**Fig. 1.**
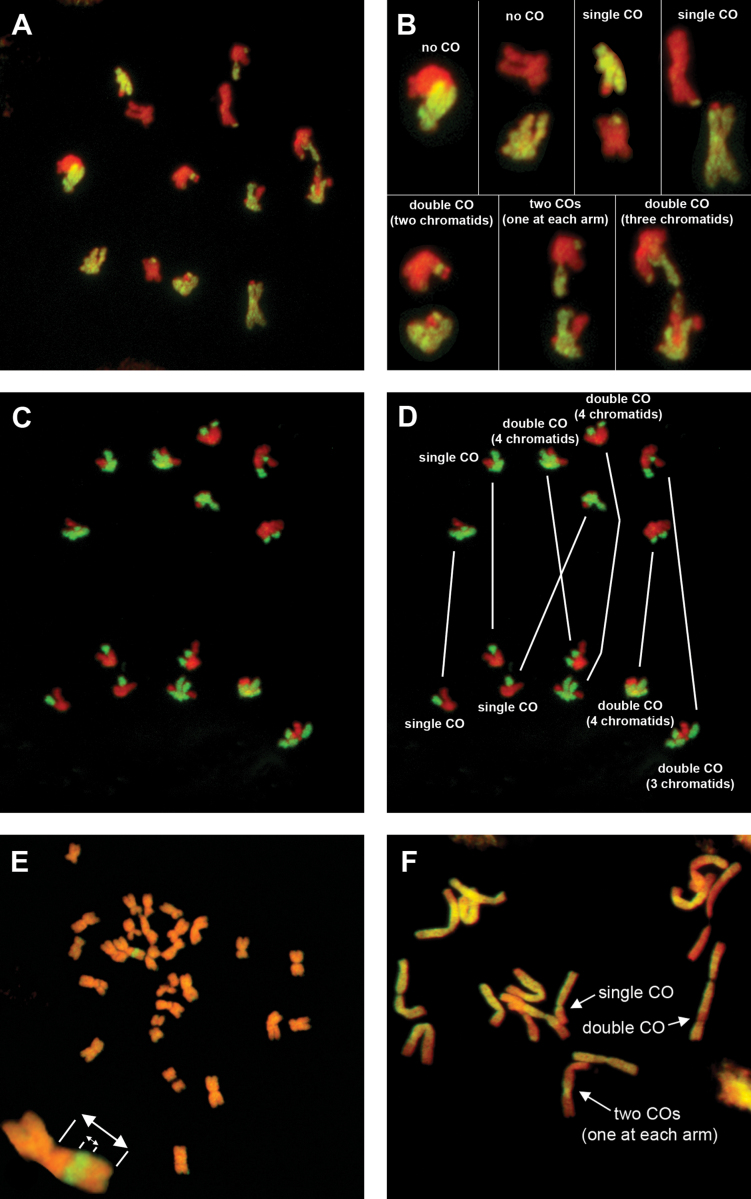
Crossovers in grasses and onions visualized by genomic *in situ* hybridization. Various types of crossovers can be seen directly during anaphase I in F_1_ hybrids of *Lolium multiflorum*×*Festuca pratensis* (A–D), in the mitotic cell of the progeny of *F. pratensis*/*L. multiflorum* single chromosome substitution lines (E), and in the mitotic cell of the progeny of F_1_*Allium cepa*×*A. roylei* hybrids (F). Insets provided in the upper right figure (B) are enlargements of the pairing partners in the metaphase I plate (A) with the description of crossover type. Similarly, white lines indicate pairing partners in the duplicated figure (D) of the metaphase plate (C). Total gDNA of *F. pratensis* and *A. roylei* was labelled with digoxigenin (green/yellow colour) and sheared DNA of *L. multiflorum* and *A. cepa* was used as blocking DNA (red pseudocolour).

**Fig. 2.**
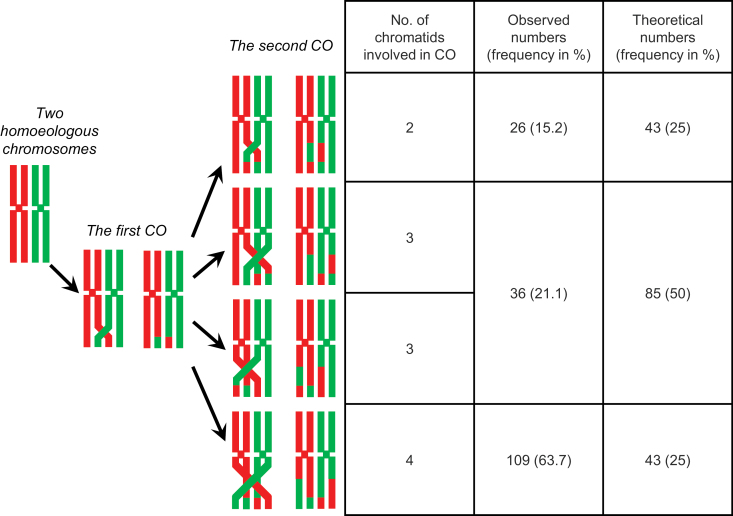
Theoretical and observed numbers and frequencies of double crossover types in diploid F_1_*L. multiflorum×F. pratensis* hybrids. Two, three, and four chromatids can be involved in a double crossover with theoretical proportions of 1:2:1 (assuming no chromatid interference).

Each pair of chromosomes or chromosome arms involves two pairs of sister chromatids. When two crossovers occur, two, three, or four chromatids can be involved (Fig. 1). With a random choice of chromatids, a 1:2:1 ratio is expected for two, three, or four chromatid involvement. In total, 171 pairs of chromosome arms with double crossovers were observed (133 + 26 + 6×2) in *Lolium×Festuca* hybrids. Four chromatids were involved in 109 double crossovers (63.7%), three chromatids in 36 (21.1%), and two chromatids in 26 (15.2%) (Fig. 2). This is significantly different from the theoretical ratio of 1:2:1 for random choice of chromatids (χ ^2^=137.9, df=2, *P*<0.001). This is based on a genome-wide analysis as identification of individual chromosomes was not possible in this experimental system.

### Crossover interference extends over ~30% of a chromosome arm length

Among the progenies of monosomic single chromosome substitution lines of *F. pratensis* into *L. multiflorum* (27 *Lolium* chromosomes and one *Festuca* chromosome) for each of the seven *Festuca* chromosomes (referred to as ‘grasses’), we scored the frequencies of single and double crossovers following their transmission to progeny. The same analysis was done in the F_2_ generation of the *Allium cepa*×*A. roylei* hybrids (referred to as ‘onions’). Single chromosome substitution lines of grasses enabled scoring of crossovers in individual chromosomes; in onion hybrids, only a genome-wide analysis was possible. Among the progenies, three types of recombined chromosomes were observed: (i) single exchanges per chromosome; (ii) double exchanges per chromosome, one in each arm; and (iii) double exchanges in one arm. It should be mentioned that the segregation of chromosomes in anaphase I and sister chromatids in anaphase II separates the products of double crossovers. Consequently, only those double crossovers involving two of four chromatids and one half of those with three chromatids can be tracked in the progeny and are considered in the subsequent analysis. Conversely, all four products (chromatids) of a double crossover where all four chromatids are involved appear in the progeny as single events and, thus, are placed in the category of single crossovers.

Out of 629 and 328 recombined chromosomes of grasses and onions, respectively, the majority demonstrated single crossovers (63.8% and 65.8%), followed by two crossovers, one on each arm (23.2% and 21.7%) and double crossovers, sometimes with single or double crossover on the other arm (13.0% and 13.1%), respectively. The proportions of chromosomes in each of the three categories varied for individual *Festuca* chromosomes (Table 1).

**Table 1.** Numbers or chromosomes with and without homoeologous crossovers and frequencies (in parentheses) of different crossover types among the progeny of single chromosome substitution lines of *F. pratensis* into *L. multiflorum* (individually for each chromosome)

Fp/Lm chromosome	No crossover	Single crossover (%)	Two crossovers, one on each arm (%)	Double crossover (%)
**1**	5	63 (63)	21 (21)	16 (16)
**2**	7	59 (57)	24 (23)	21 (20)
**3**	4	58 (62)	25 (27)	10 (11)
**4**	6	56 (70)	18 (23)	6 (8)
**5**	3	62 (78)	10 (13)	7 (9)
**6**	2	26 (40)	27 (42)	12 (18)
**7**	6	67 (77)	10 (11)	10 (11)
**Average**		**(64)**	**(23)**	**(13)**

**Fig. 3.**
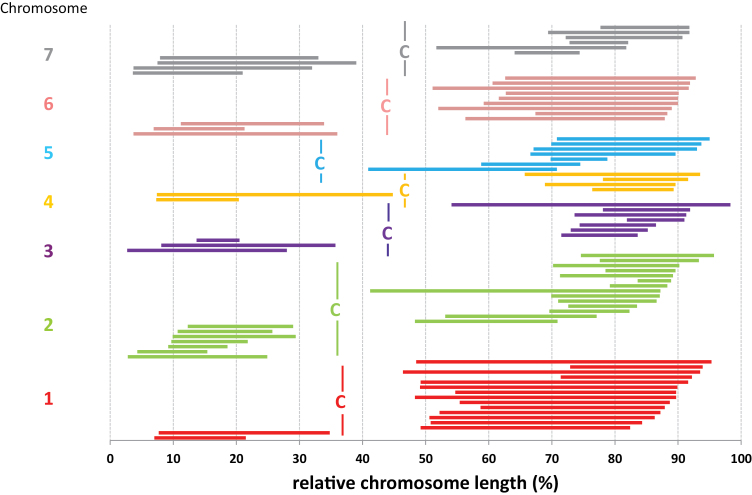
The distribution of double crossovers along individual chromosomes of *F. pratensis*/*L. multiflorum*. Short arms are on the left and long arms on the right. The coloured lines represent the recombined segments in all chromosome arms (different colours). The position of the centromere in each chromosome is indicated by a vertical line with letter ‘C’.

Using progenies of single chromosome substitution lines, it was possible not only to estimate the frequencies of double crossovers for each of the *Festuca* chromosomes, but also to estimate the mean distances in megabases between adjacent crossovers in each chromosome (Fig. 3; Table 2). The frequencies of double crossovers differed markedly between the short and long arms. In general, the short arms had considerably lower frequencies of double crossovers compared with the long arms. In the shortest arm of the *F. pratensis* karyotype, that of chromosome 5, we did not observe any products of double crossover (involving two or three chromatids). With the obligatory chiasma and the positive crossover interference, this is a typical distribution as affected by the chromosome arm length. It was clearly evident in wheat during the genetic mapping of the physical attributes of chromosomes (Lukaszewski and Curtis, 1993), and was confirmed by ultra-high-resolution analysis (Jordan *et al.*, 2018).

**Table 2.** Average distances between two events spanning double crossover for individual *F. pratensis*/*L. multiflorum* chromosomes/chromosome arms

Fp/Lm chromosome	Length (µm^*a*^)	1C (Mb^*a*^)	Average intercrossover distance and the range (in parenthesses; in Mb)
1	4.67	373	122
1S	1.71	137	77 (55–100)
1L	2.96	237	129 (74–168)
2	6.07	485	77
2S^*b*^	2.42	194	72 (49–99)
2L	3.64	291	80 (26–147)
3	6.25	499	90
3S^*c*^	2.92	233	97 (35–134)
3L	3.33	266	87 (45–221)
4	6.79	543	112
4S	3.18	254	139 (70–209)
4L	3.61	289	99 (62–149)
5	5.04	403	79
5S	1.76	140	–
5L	3.29	263	79 (43–102)
6	4.93	394	107
6S	1.97	158	85 (54–118)
6L	2.95	236	115 (84–147)
7	6.05	484	97
7S	2.90	231	123 (89–145)
7L	3.16	252	80 (42–134)
**Average**			**97.2**

^*a*^ 1C=molecular size of one copy of individual chromosome/chromosome arm; adopted from Kopecký *et al.* (2010).

^*b*^ Chromosome arm with 5S rDNA.

^*c*^ Chromosome arm with 45S rDNA.

On average, two adjacent crossover events on one arm of a single chromatid were separated by ~97 Mb. However, this distance showed considerable variation from one chromosome arm to another. The shortest distance between two adjacent crossovers in an arm was ~26 Mb. There was no correlation between chromosome/chromosome arm length (CL), the frequency of double crossover (FdCO), and the average distance (AD) between two crossovers on one arm (double crossovers involving two or three chromatids, but not four) as revealed by Spearman correlation coefficient (for arms, *n*=14: CL versus FdCO *r*=0.42, *P*=0.149; CL versus AD *r*=0.19, *P*=0.535; FdCO versus AD *r*= –0.15, *P*=0.635; for chromosomes, *n*=7, CL versus FdCO *r*= –0.43, *P*=0.333; CL versus AD *r*= –0.29, *P*=0.535; FdCO versus AD *r*= –0.20, *P*=0.670).

**Fig. 4.**
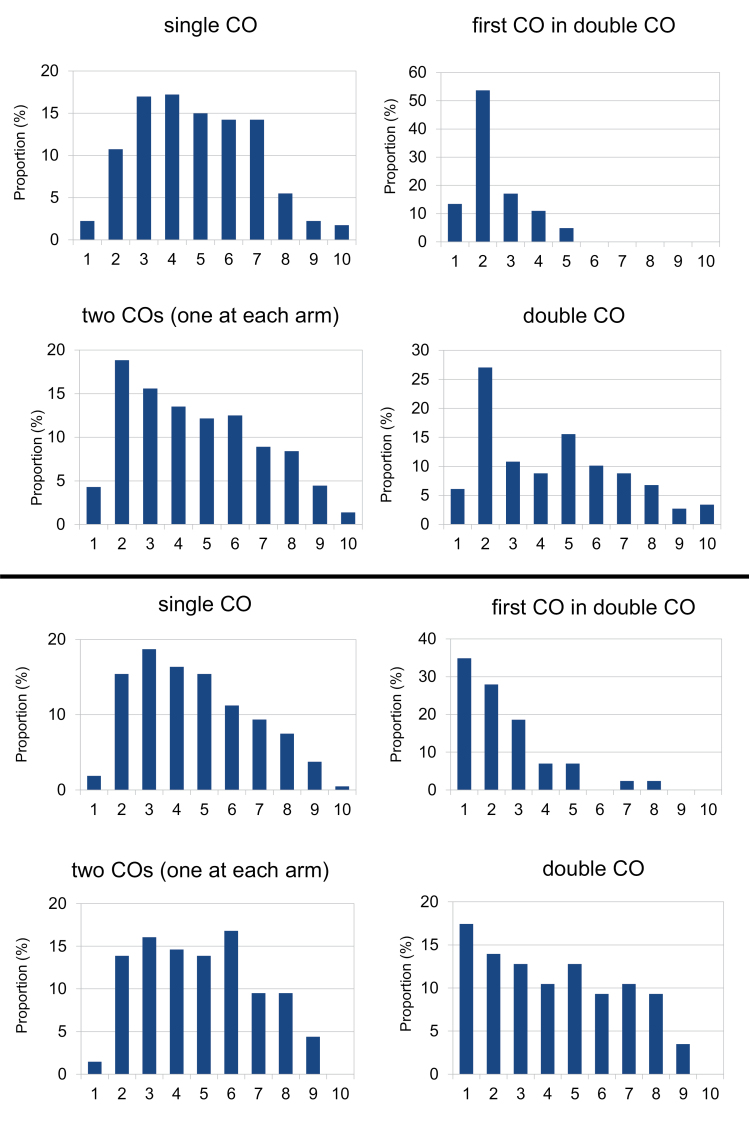
The frequency and distribution of crossovers based on their types in grass (upper part) and onion (lower part) hybrids. The *x*-axis represents a chromosome arm (from the telomere on the left to the centromere on the right) divided into bins of 10% of relative arm length.

The distribution of single and double crossovers differed in both grass and onion hybrids (Fig. 4). The distribution of single crossovers in both hybrids showed similar patterns, with the highest frequency in the intervals from 20% to 40% of the arm length from the telomeres, and with a significant decline in frequencies nearing the centromeres (proximal 30% of the arm). Relatively low frequencies were also evident in the most distal 10% of the arm (next to telomeres). However, this may be an artefact of the method used, as short terminal segments from the non-probe parent may be undetectable by *in situ* probing with labelled DNA (Lukaszewski *et al.*, 2005). On the other hand, in grasses, double crossovers had the highest frequency in the intervals of 10–20% (the first crossover) and 40–50% (the second crossover) of the arm measured from telomeres. This indicates that crossover interference in grasses acts up to a distance of ~30% of the average arm length. A similar crossover distribution was observed in onions, even though no clear frequency peaks were evident. Once we calculated the frequencies of the theoretical and actual appearance of double crossovers for all combinations of intervals (an interval equalled 10% of the physical arm length), the positive crossover interference was observed between intervals 0–10% and 10–20%, 10–20% and 20–30%, 10–20% and 30–40%, 20–30% and 30–40%, 20–30% and 40–50%, 20–30% and 50–60%, 30–40% and 40–50%, and between 30–40% and 50–60% (data not shown). On average, two crossovers in one arm were separated by 36% and 41% of the arm length (ranging from 13% to 78% and from 9% to 83%) in onions and grasses, respectively (Fig. 4). This supports the indication that in both hybrids, crossover interference acts at a physical distance of 30–40% of an average chromosome arm. This does rule out the possibility that the crossover interference is governed by mechanisms modulated by the absolute physical distance between two crossovers.

In general, the average frequency of chromosomes with double crossovers in grasses was 13% (Table 1). The highest frequency of such events was ~20% for chromosome 2, which also had the lowest average distance between two adjacent crossovers (77 Mb) (Table 2), and the lowest was 8% for chromosome 4, which is the longest chromosome in the genome (Kopecký *et al.*, 2010) and seems to have strong crossover interference (average length between two events spanning a double crossover was 112 Mb) (Table 2). On the other hand, chromosome 1 is the shortest in the *F. pratensis*/*L. multiflorum* genomes (Kopecký *et al.*, 2010) but it showed the highest average distance between adjacent crossovers. This suggests that the interference distance in the system studied here may be chromosome specific and not genome wide.

There was a significant difference in the distribution of single crossovers along the chromosome arms and of the first crossover when two crossovers were present on an arm, both in grasses (Kolmogorov–Smirnov test, D=0.542, bootstrap *P*<0.001) and in onions (D=0.455, bootstrap *P*<0.001) (Fig. 4). When two crossovers were present, the first crossovers were much more distal, with a peak at 10–20% of the arm length, and dropped rapidly towards the centromere. In grasses, the first crossovers of double crossover arms were limited to the distal half of the arms. A similar distribution was observed in onions, with a gradual decrease from the telomere toward the centromere. It is evident that second crossovers on an arm are possible only when the first crossover is sufficiently distal.

### Crossover interference does not act across the centromere

To test if crossover interference acts across the centromere, or if the two arms of a chromosome are independent for crossover formation, we performed three comparisons between the expected values and the experimental data. If the interference acts across the centromere, the following should apply. (i) The distribution of single crossovers and of two crossovers (each on one of the arms) should differ, with the latter located more distally towards the telomeres. Indeed, we observed that the distribution of single crossovers per chromosome and two crossovers per chromosome with one at each arm differed significantly in grasses (Kolmogorov–Smirnov test, D=0.101, bootstrap *P*=0.002), but not in onions (D=0.079, bootstrap *P*=0.334). In grasses, however, single crossovers were distributed primarily in the interstitial parts of the chromosome arms, while two crossovers were more spread over the arms and were localized relatively frequently outside the interstitial regions. In onions, single crossovers and two crossovers were spread over the arms, except for a rapid decline in the centromeric and pericentromeric regions. (ii) When a double crossover is formed on one arm, the distribution of crossovers on the other arm should be more distal. Although there were not enough such cases for statistical tests, the average positions of crossovers were similar between a single crossover, two crossovers (one on each arm), and a single crossover with a double crossover on the other arm (Table 3). (iii) With two crossovers in one arm, there should be fewer crossovers in the other arm as compared with a single crossover in the first arm. No difference was observed in the distribution of crossovers in the second arm when comparing one and two crossovers in the first arm in both grasses (Fisher’s exact test, *P*=0.415) and onions (Fisher’s exact test, *P*=0.754) (Table 4).

**Table 3.** Average positions of crossovers based on their abundance (in % of the arm length, measured from the telomere)

Type of crossover	Average position of crossover (% of arm from telomere)	
	Onions	Grasses
Single crossover	41.32	43.5
Two crossovers (one on each arm)	43.85	40.6
Single crossover with double crossover on the other arm	46.49	38.6

**Table 4.** The relationship between the number of crossovers in one chromosome arm and the number of crossovers in the other arm

	**Grasses**			
No. of crossovers on one arm	Number of crossovers on the other arm			
	0	1	2	3
0		308	133	0
1	308	66	26	0
2	133	26	6	0
3	0	0	0	0
	**Onions**			
No. of crossovers on one arm	Number of crossovers on the other arm			
	0	1	2	3
0		215	31	2
1	215	57	9	1
2	31	9	0	0
3	2	1	0	0

These three tests indicate that in grasses and in onions, crossover interference does not act across the centromere; two arms of a single chromosome appear to be independent for establishment of the crossover.

## Discussion

This study was performed on wide hybrids and so observations and results may not be fully representative of strictly homologous chromosome pairing and recombination. Wide hybrids made direct observations possible and offered a unique chance of studying the immediate effects of crossing over as early as anaphase I, but at the same time they might have skewed the results in some unpredictable direction. However, these hybrids show surprisingly high regularity of meiotic chromosome pairing and segregation (Jauhar, 1975; Kopecky *et al.*, 2008), suggesting that the results may be of wider significance. On the other hand, there is a chance that the distribution of crossovers, as judged by the exchange points in recombined chromosomes, may be wider than what could be expected based on the chiasma distribution in the parental species (Karp and Jones, 1983). It also must be pointed out that data on the physical distribution of crossovers along chromosomes and chromosome arms do carry an inherent error. There are differences in genome sizes of the parental species of both hybrids, and these translate into differences in chromosome lengths. Whether these differences are evenly distributed along all chromosomes is very far from clear. For that reason, most of the analyses are based on relative length values.

### Chromatid interference

The ability to distinguish parental chromatin by chromosome painting allowed us to analyse the frequency and distribution of crossovers directly during the meiotic division, immediately after bivalent separation, and ensured that all products of every individual meiosis could be scored (Fig. 1). This avoided any possible bias associated with uneven chromosome transmission and gametic or zygotic selection. These direct observations were supplemented with earlier observations of the structure of chromosomes among the progeny.

Ever since Barbara McClintock’s demonstration of the relationship between chiasmata and crossing over (Creighton and McClintock, 1931), chiasmata alone indicated that crossovers were not distributed evenly along chromosomes. This has been demonstrated in many organisms and in several different ways (reviewed in Otto and Payseur, 2019). The pattern of crossovers is affected by genetic and presumably epigenetic systems, with a general preference for distal location, and perhaps by the chromatin structure, which makes some parts of the chromosomes inaccessible to crossing over, regardless of the position on the telomere–centromere axis (Lukaszewski, 2008; Lukaszewski *et al.*, 2012; Higgins *et al.*, 2014). Within this general pattern, if more crossovers are formed in an arm, their frequencies and distribution tend to form a sinusoid pattern, reflecting the presence and strength of the positive crossover interference (Muller, 1916; Mather, 1938).

Of the two components of genetic interference, crossover interference can be easily scored in any genetic mapping experiment. The second component, chromatid interference, is far more difficult to observe. It informs if the chromatids involved in the first crossover affect the choice of chromatids for a second crossover on the same chromosome. Chromatid interference can only be reliably and directly scored when all four products of a single meiosis are available, and this happens infrequently. Consequently, the current knowledge of chromatid interference stems from statistical models, and the general assumption is that chromatid interference does not exist. Geneticists generally assume its absence while studying recombination and creating genetic maps (Strickland, 1958; Broman and Weber, 2000; Broman *et al.*, 2002).

Early studies on *Neurospora crassa*, *Saccharomyces cerevisiae*, and *Aspergillus nidulans* indicated that the choice of chromatids for second crossovers may not be random (Lindegren and Lindegren, 1942; Hawthorne and Mortimer, 1960). Similarly, the model of Teuscher *et al.* (2000), based on data from a study on mouse and two studies on *Drosophila*, suggested that chromatid interference may play an important role in meiosis. Similarly, weak chromatid interference was deduced in maize, human oocytes, and *Caenorhabditis elegans* (Hou *et al.*, 2013; Li *et al.*, 2015; Li *et al.*, 2018). Our study offers clear and unambiguous evidence for a strong positive chromatid interference: the second crossover in an arm was formed more frequently between chromatids not involved in the first crossover (Fig. 2). In other words, all four chromatids of two homoeologous chromosomes were involved in double crossovers much more frequently than expected (64% versus 25%) and the most abundant class expected for random chromatid selection, that with three chromatids involved, was seriously under-represented, accounting for only 21% of cases versus 50% of those theoretically expected. Thus, our results provide strong support for the model of Teuscher (2000) and deliver interesting results for further studies on crossover formation and recombination in plants. They also point out a need to consider chromatid interference in genetic mapping. We envisage that newly developed visual assays for yeasts and the model plant Arabidopsis (Berchowitz and Copenhaver, 2008), which enable scoring all four products of meiosis, could increase our understanding of chromatid interference in the near future.

The molecular mechanism underlying chromatid interference is unknown. However, a study on *C. elegans* suggests that an orthologue of the breast cancer susceptibility gene 1 (*BRCA-1*) may be involved in this pathway. Numbers of double crossovers with two chromatids involved were reduced and numbers of single crossovers were elevated in the *brc-1*;*zim-1* mutant compared with the *zim-1* single mutant. This indicates that BRCA-1 may counteract chromatid interference under meiotic dysfunction, such that more crossovers with the same two chromatids involved occur (Li *et al.*, 2018). Some studies also pointed to the role of Tel1, a protein kinase that responds to DNA damage, in both crossover and chromatid interference (Zhang *et al.*, 2011; Garcia *et al.*, 2015; Cooper *et al.*, 2016). According to Fowler *et al.* (2018), Tel1 is activated when the first formation of DSBs occurs in a clustering hotspot, promoting the phosphorylation and inactivation of some components of the DSB-forming complex, which prevents the formation of a second DSB in the vicinity. In the absence of Tel1, multiple breaks are formed, increasing the frequency of crossovers. The clustering of DSB hotspots can involve each homologous chromosome separately (two chromatids) or both together (four chromatids), and in species where clustering involves the homologues pair the interference is stronger (Smith and Nambiar, 2020). As such, cluster gathering of four chromatids may explain the chromatid interference found in our study.

### Crossover interference

Apart from a few exceptions, such as *A. nidulans* and *S. pombe* discussed above, crossover interference is a widespread phenomenon in eukaryotes (Strickland, 1958; Snow, 1979), but its strength and spatial efficiency differ among species. In roundworm *C. elegans*, the absence of double crossovers may be taken as an indication of complete crossover interference (Meneely *et al.*, 2002). Similarly, crossover interference appears to be extremely strong in mouse (Broman *et al.*, 2002). In other species, it may be strong in short intervals: it is almost complete in *D. melanogaster* and very strong in *Neurospora crassa* (Foss *et al.*, 1993).

Similar to genetic studies based on screening of progeny to assess the crossover interference, our approach did not allow incorporation of a double crossover where four and three chromatids were involved. Thus, it provides detailed characterization of crossover interference arising exclusively from double crossovers involving two chromatids. In grasses and onions, we never observed double crossovers at a distances shorter than 9% and 12% of the arm length, respectively. Using genome-wide numbers, this translates to ~37 Mb and 241 Mb (Labani and Elkington, 1987; Doležel *et al*., 1992; Kopecký *et al.*, 2010). As the distance from the initial crossover increases, the interference strength weakens and chances for a second crossover increase. Crossover interference is effective up to 15–20 Mb in *Drosophila*, corresponding to about one half of a chromosome arm, and up to 0.9 Mb in *Neurospora*, corresponding to ~30% of an average arm length (Foss *et al.*, 1993). Similarly, Lawrie *et al.* (1995) observed crossover interference extending over ~68% and 77% of a chromosome length in mouse autosomes 5 and 15, corresponding to about one-third of the arms. Complete crossover interference over distances equalling 25–30% of a chromosome arm has been confirmed in *Chorthippus branneus* (Jones, 1987). In budding yeast, Malkova *et al.* (2004) detected interference over distances of about a quarter of the total length of a chromosome arm.

The examples listed above indicate that the effectiveness of crossover interference is surprisingly similar among the species tested, and it extends up to about one-third of a chromosome arm. Even though we worked with wide hybrids, the results follow the same pattern: in both cases, crossover interference acts efficiently up to ~30–40% of the chromosome arms (Fig. 3). The same range was observed in the B genome of wheat (Lukaszewski and Curtis, 1993). Interestingly, it appears that it is the relative distance along the chromosome arm and not the physical or genetic distance that matters. The two hybrids used in our study differ significantly in their genome sizes and lengths of chromosomes in megabases: an average chromosome in onions is ~2 Gb in length, while it is ~0.4 Gb in grasses (Doležel *et al*., 1992; Kopecký *et al.*, 2010), corresponding to a physical crossover interference distance of ~350 Mb and 100 Mb, respectively (Table 2). Mean interchiasma distances in mouse and *S. cerevisiae* chromosomes gave the impression of being correlated with chromosome lengths so that the mean interchiasma distance was greater in longer chromosomes (Lawrie *et al.*, 1995). The possible mechanism explaining this phenomenon involving a conformational chain reaction leading to the allosteric blocks of recombination in neighbouring regions in a time-dependent manner has been described by Kaback *et al.* (1999). This indicates that the processes underlying crossover interference may be evolutionarily conserved. We should note that despite the general mechanism of crossover interference acting at the whole-genome level, there are exceptions where crossover interference has variable intensities and distributions in different regions of a chromosome, such as observed in budding yeast and in *Populus euphratica* (Malkova *et al.*, 2004; Wang *et al.*, 2019).

As in other cases studied earlier (see John, 1990), we observed that second crossovers on an arm are predominantly formed only when the first crossovers are sufficiently distal (Fig. 4). This may be related to the progress of synapsis, from the telomere toward the centromere, with the distal crossover (presumably formed first) leaving enough room on the arm for crossover interference strength to drop before the end of the recombination process, hence increasing the opportunity for an additional crossover to form. The role of the centromere as a barrier for crossover interference remains unclarified (reviewed in Nambiar *et al.*, 2019). The question is: are the two arms of a chromosome independent in crossover formation or can a crossover on one arm interfere with crossovers on the other arm? The current evidence is unclear. No positive interference was observed across the centromere in *N. crassa* (Strickland, 1961; Perkins, 1962). On the other hand, in the grasshopper, Colombo and Jones (1997) provided evidence that interference did act across the centromere, and the level of interference in the region spanning centromeres appeared to be no different from that seen in any other chromosome segment. Similarly, the chiasmata numbers on two arms of a single chromosome do not appear to be independent in humans (Broman and Weber, 2000) or in species such as *Culex* and *Paeonea* (Callan and Montalenti, 1947; Harte, 1956). In this study, we did not observe any significant effect of crossovers in one arm on the crossover frequency and distribution in the other arm, implying that in our material crossover interference may not extend across the centromere (Tables 3, 4). However, crossover distribution might have also played a role here: with predominantly distal crossovers, the distance across the centromere in most cases would be greater than the interference distance.

In conclusion, our study clearly illustrates that chromatid interference does operate in higher Eukaryotes, along with crossover interference. Both play an important role in meiotic division, the latter acting over a distance corresponding to 30–40% of the physical length of a chromosome arm. In materials studied here, the centromere appears to act as an effective barrier for crossover interference and thus two arms of a chromosome seem to act as independent units for crossovers. We are aware that our study is based on observations of crossovers between homoeologous chromosomes in wide hybrids and these may not completely correspond to interactions between perfectly homologous chromosomes. However, meiotic chromosome pairing in these hybrids is essentially normal (for illustrations of meiosis in the *Lolium×Festuca* hybrid see https://olomouc.ueb.cas.cz/getattachment/Research-groups/Kopecky-group/Meiosis-With-Labeled-Parental-Genomes.pdf.aspx?lang=en-US). Moreover, all crossovers must meet certain DNA criteria, both for the substrate length and for the level of homology (Shen and Huang, 1986, 1989; Datta *et al.*, 1997) and there is no reason to suspect that the crossovers observed here violate them. Given the recent progress of technology, we envisage that it will be possible to monitor both homologous and homoeologous crossovers in the near future using tetrad analysis based on the *quartet1* (*qrt1*) mutation, haplotype-specific chromosome painting as shown in maize, or the DeepTetrad technology currently available only for *Arabidopsis thaliana* (Copenhaver *et al.*, 2000; Martins *et al.*, 2019; Lim *et al.*, 2020).

## Data Availability

All data supporting the findings of this study are available within the paper.
